# Outcomes of Budesonide as a Treatment Option for Immune Checkpoint Inhibitor-Related Colitis in Patients with Cancer

**DOI:** 10.3390/cancers16101919

**Published:** 2024-05-18

**Authors:** Antonio Pizuorno Machado, Abdullah Salim Shaikh, Alice Saji, Malek Shatila, Isabella Glitza Oliva, Yinghong Wang, Anusha Shirwaikar Thomas

**Affiliations:** 1 Department of Internal Medicine, The University of Texas Health Science Center, Houston, TX 77030, USA; 2 Department of Gastroenterology, Hepatology, and Nutrition, The University of Texas MD Anderson Cancer Center, Houston, TX 77030, USAmshatila@mdanderson.org (M.S.); icglitza@mdanderson.org (I.G.O.); ywang59@mdanderson.org (Y.W.)

**Keywords:** immune checkpoint inhibitors, immune mediated colitis, budesonide

## Abstract

**Simple Summary:**

Guidelines from oncologic societies recommend high-dose systemic corticosteroids as the primary therapy with or without biologics for moderate to severe colitis secondary to immune checkpoint inhibitors; budesonide is a gut selective steroid that undergoes first past metabolism with less systemic adverse events in comparison to systemic steroids, although its use has not been studied or established for colitis secondary to immune checkpoint inhibitors. Budesonide employed for other gastrointestinal diseases, such as inflammatory bowel disease, is associated with decreased infections and metabolic side effects. In our analysis, we observed that budesonide may be an effective strategy to treat and prevent the recurrence of colitis with similar results as systemic corticosteroids in this immunocompromised cancer patient population.

**Abstract:**

Background: Current treatment guidelines for moderate to severe colitis (IMC) secondary to immune checkpoint inhibitors (ICI) recommend systemic corticosteroids as the primary therapy in conjunction with biologics, namely infliximab and/or vedolizumab. We aimed to explore the efficacy and safety of oral budesonide in the treatment of IMC. Methods: We performed a retrospective analysis at MD Anderson Cancer Center of adult cancer patients with a confirmed (based on clinical, radiographic and laboratory assessment) diagnosis of IMC between 1 January 2015 and 31 November 2022, treated with budesonide. Data collection included demographics, oncologic history, IMC-related information and outcomes up to 6 months after the last dose of ICI. Results: Our sample (n = 69) comprised primarily of Caucasian (76.8%) females (55.1%). The majority of patients received combination therapy with anti-PD-1/L1 and anti-CTLA-4 (49.3%), and the most common malignancy treated was melanoma (37.6%). The median grade of diarrhea was 3 and of colitis was 2. Of the 50 patients who underwent endoscopic evaluation, a majority had non-ulcerative inflammation (64%) and active colitis on histology (78%). Budesonide was used as primary treatment at onset of IMC in 56.5% patients, as well as a bridging therapy from systemic corticosteroids in 33.3%. Less than half of the patients (44.9%) required additional therapies such as biologics or fecal microbiota transplant. Additionally, 75.3% of patients achieved full remission of IMC and 24.6% had a recurrence of IMC. ICI was resumed in 31.9% of patients and 17.4% received other forms of cancer therapies. Conclusions: Budesonide may be an effective strategy to treat and prevent the recurrence of IMC. The remission rates observed in our analysis with budesonide alone are comparable to systemic corticosteroids. Patients that require an extended duration of steroid exposure and those with moderate to severe colitis may benefit from budesonide given its lower risk of infection and complications. Furthermore, we observe that budesonide may serve as a successful bridge from systemic corticosteroids with subsequent biologic treatment. Larger prospective studies are necessary to determine the role of budesonide as well as its safety profile.

## 1. Introduction

Immune checkpoint inhibitors (ICIs) are paramount in the treatment of various malignancies. However, while conferring an appreciable survival benefit, these agents also predispose patients to unique immune-related adverse events (irAEs), with diarrhea and colitis amongst the most common toxicities reported [1]. Immune-mediated colitis (IMC) has been reported in up to 40% of patients treated with ICIs, varies widely in severity [2,3], and can be a cause for discontinuation of ICI therapy [4]. Failure in early recognition and delayed or suboptimal treatment can lead to an increased risk of complications such as bowel perforation [5].

The management of IMC involves the use of systemic steroids with selective immunosuppressive therapy (e.g., infliximab, vedolizumab) for CTCAE (grade of diarrhea) [6] grade 2 or higher toxicity which has been found to achieve response rates of as high as > 80% [7,8]. Ustekinumab, tofacitinib, and fecal microbiota transplantation are also reported to be effective [9,10,11] in treating steroid-refractory cases.

Inflammatory bowel disease (IBD) and IMC share multiple similitudes, such as the disruption in the immune surveillance pathways and gut homeostasis and consequent acute and/or chronic gastrointestinal inflammation [9]. The clinical and endoscopic presentation of IMC is highly reminiscent of IBD and therefore, when considering the type of available treatments for IMC, one can infer that similar options are routinely used and recommended for IBD. One suggested treatment is budesonide, which has been shown to be efficacious in the induction of remission in IBD [12,13,14,15,16].

Budesonide is an enteric-coated synthetic glucocorticoid which is absorbed in the distal small bowel where it undergoes extended first-pass metabolism from cytochrome P450 3A4 in the liver to compounds with negligible glucocorticoid activity (16α-hydroxyprednisolone and 6β-hydroxybudesonide) [17] making it gut selective in action with minimal systemic side effects, in stark contrast to systemic corticosteroids. While a mainstay of IMC treatment, systemic steroids unfortunately bear several side effects including immunosuppression, adrenal suppression, weight gain, high blood pressure, hyperglycemia, etc. [18] They also increase the potential risk for opportunistic infections and negatively affect anti-tumor immunity [19]. Systemic immunosuppression is especially concerning as it could potentially worsen one’s response to ICI treatment and cancer prognosis. The use of budesonide as an alternative to systemic steroids for treating IMC could theoretically mitigate these risks while providing efficacious treatment for IMC; the use of budesonide for IMC was observed in a 39-patient cohort in which 13 patients received budesonide versus 25 receiving systemic steroids with no difference in terms of time from symptom onset to resolution and increased ICI cycles after being diagnosed with IMC [20].

However, the use of budesonide for IMC is currently limited to less severe cases as recommended by the European Society of Medical Oncology guidelines [21]. There is no available data on its role for long-term prophylaxis and/or as a bridge from systemic corticosteroids. Therefore, in this study, we aimed to study the efficacy and safety of budesonide as a therapy for IMC.

## 2. Methods

### 2.1. Study Design and Population

**Figure 1:**
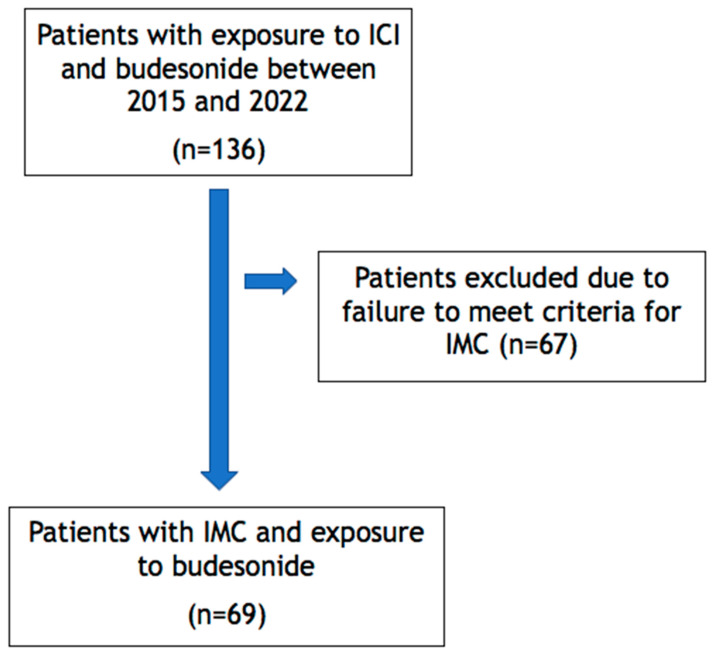
Patient selection diagram.

This retrospective chart review is a descriptive, single-center study that included adult patients who were diagnosed with IMC and treated with budesonide (9 mg daily) at a tertiary cancer center between 1 January 2015 and 31 November 2022. This study was approved by the institutional review board with a waiver of patients’ informed consent. We identified adult cancer patients 18 years or older who (1) were treated with ICIs for various types of cancer, (2) had a diagnosis of immune-mediated colitis at least 6 months after the last ICI dose, and (3) received treatment with budesonide. Patients with pre-existing inflammatory bowel disease (IBD), microscopic colitis, or other autoimmune gastrointestinal disorders were excluded (Figure 1).

### 2.2. Clinical Data

Demographic variables and cancer-related information such as age, gender and race were tabulated. Similarly, oncologic variables such as primary cancer type, stage, cancer treatments and doses received as well as Charlson Comorbidity Index score were collected. The diagnosis of colitis was based on the clinical presentation, endoscopic and histologic features after exclusion of other etiologies. The colitis-related data presented includes time of onset after exposure to ICI, cycles of ICI before colitis, type of ICI, and peak Common Terminology Criteria for Adverse Events (CTCAE) [1] grades for colitis and diarrhea. Detailed information regarding treatment for colitis such as steroids, infliximab, and vedolizumab as well as presentation on lower endoscopy and pathology at the time of colitis diagnosis were collected.

### 2.3. Statistical Analysis

The statistical analyses performed were descriptive in nature. The distributions of continuous variables were summarized by medians and interquartile ranges. The distributions of categorical variables were summarized by frequencies and percentages. These were calculated using SPSS 26.

## 3. Results

### 3.1. Patient Population, Characteristics and Oncologic History

Our sample comprised 69 patients that met the inclusion criteria. The included patients had a median age of 64 years with 31 patients (44.9%) being male and 53 patients (76.8%) being white (Table 1).

As for oncological history, 26 patients (37.6%) were diagnosed with melanoma, followed by lung/head and neck cancers in 12 patients (17.3%), genitourinary cancer in 11 patients (15.9%), and gastrointestinal in 6 patients (8.6%). The majority of the patients (n = 55, 79.7%), had stage IV cancer. With regard to type of ICI, 34 (49.3%), 28 (40.6%) and 7 (10.1%) patients received a combination of PD-1/L1 and CTLA-4, PD-1/L1 inhibitor monotherapy and CTLA-4 monotherapy, respectively. Patients underwent a median of three cycles of ICI prior to a diagnosis of colitis. Twenty-two (22) patients (31.8%) resumed ICI after recovering from colitis, while 12 patients (17.3%) continued other forms of cancer therapy.

### 3.2. Characteristics of Colitis

The predominant symptom of IMC was diarrhea in all 69 patients (100%), followed by abdominal pain in 65 patients (94.2%) (Table 2). The median CTCAE grade in our sample was 2 and 3 for colitis and diarrhea, respectively. The median fecal calprotectin before budesonide treatment was 671 mcg/gm (Table 2). Half of our sample (n = 35, 50.7%) was hospitalized for colitis. As for the treatment of colitis, budesonide was used alone in 38 patients (55.1%), and in conjunction with vedolizumab (n = 15, 21.7%) or infliximab (n = 7, 10.1%). Fecal microbiota transplant was performed in three patients who had received budesonide and biologic therapy and had refractory disease course (4.3%) (Table 3). Overall, in comparison, the characteristics of IMC and endoscopic features of budesonide-only treated patients was similar to the whole cohort.

### 3.3. Endoscopic- and Histology-Related Characteristics

At the time of diagnosis of IMC, 50 patients underwent an endoscopic procedure. Non-ulcerative inflammation was the predominant endoscopic finding in 32 patients (64%) followed by ulcerative inflammation (18%) and normal appearing colonic mucosa (18%). On histology, the majority had active inflammation (78%) (Table 3).

### 3.4. Budesonide Use Characteristics

Budesonide was used in different strategies in patients with IMC. Thirty-nine (39) patients received budesonide primarily to treat colitis (56.5%) and 23 patients (33.3%) received budesonide after being on systemic steroids, such as prednisone, for the treatment of IMC as a transition. Four (4) patients received budesonide for a longer term as secondary prophylaxis to prevent IMC recurrence on rechallenge of cancer therapy. Three (3) of our patients received budesonide for other immune-related adverse events. The dose of budesonide was at 9 milligrams daily. The median duration of budesonide therapy was 42.5 days with 51 patients (75.3%) achieving remission by the end of our study period. Recurrence of colitis was noted in 17 patients (2.6%) (Table 4).

### 3.5. Budesonide Safety Profile

Five (5) (7.3%) patients were found to have an infection about 2 months after exposure to budesonide. Six (6) (8.7%) developed adrenal insufficiency or need for long-term hydrocortisone. Two (2) patients developed new onset diabetes mellitus. See Table 5 for details. As for the comparison of patients that received steroids before budesonide and those treated primarily with budesonide, it was shown that overall, the incidence of infection was similar, although the group treated with steroids before budesonide had a higher rate of ICU admission and adrenal insufficiency (Table 6).

### 3.6. Cancer Status at Follow up

Twenty-nine (29) patients’ cancer status was stable (42%), 38 progressed (55%), and 2 went into remission (2.9%) (Table 5).

## 4. Discussion

IMC is the most frequently encountered toxicity secondary to immunotherapy and is often the reason for interruption in cancer care. Presently, high-dose corticosteroids form the backbone of the immunosuppressive treatment of this inflammatory toxicity that bears a striking resemblance to IBD, although additional immune-modulatory agents may be required for steroid-refractory cases, or for their steroid-sparing effect. [20,21]. However, systemic corticosteroids have a myriad of side effects which are particularly paramount in patients who are already immunosuppressed from their malignancies and cancer treatments. Our group has previously shown that the length of systemic steroid exposure can independently increase the risk of infection in a cancer patient population with a significantly higher risk when used for >30 days [8,22]. Furthermore, a recent systematic review assessing the safety profile of immunosuppressants in the management of irAEs which included 11 studies (1036 patients) showed that adverse events from irAE therapy occurred in about one-third of patients that received either systemic steroids or a combination of the same and other immunosuppressants [5]. Burdett et al. [23] showed that patients receiving steroids to treat irAEs had heterogeneous results regarding their cancer outcomes.

In contrast, budesonide bears a gut selective action in light of the drug’s first pass metabolism and pharmacokinetic profile, which significantly lowers systemic bioavailability (10–15%) [24] and the potential side effects in comparison to systemic corticosteroids [13] and this has been successfully employed in the management of IBD and autoimmune hepatitis. Studies demonstrate efficacious induction of remission with budesonide [25,26], administered orally at 9 mg daily for 6 to 12 weeks and tapered over 2 to 4 weeks. These patients were also noted to have a significantly lower adverse event rate, i.e., 37% compared to 62% in IBD patients treated with standard systemic steroids [27]. Furthermore, bacterial and most viral infections occur less in budesonide-treated patients compared to patients treated with systemic glucocorticoids [28]. These findings were redemonstrated in a systematic review and network meta-analysis of 31 trials including 5689 patients with IBD, wherein budesonide was associated with significantly fewer corticosteroid-related AEs than oral systemic corticosteroids [OR 0.25, 95% CI: 0.13–0.49] [16]. Additionally, it has been shown that while both forms of steroid can induce remission in IBD, the gut selective form poses less suppression of pituitary-adrenal function [13]. We therefore present data on the utility and safety of budesonide as a gut selective steroid in the management of these patients with IMC as an alternative to systemic corticosteroids.

Our sample was most representative of moderate severity IMC based on the CTCAE clinical grade and the majority with non-ulcerative inflammation on endoscopy (Figures S1 and S2). More than half of our sample received budesonide exclusively with or without exposure to biologics and ~75% attained remission of colitis. Notably, our data demonstrates slightly improved results, nonetheless, in keeping with previous studies wherein the remission rate of IMC with corticosteroid use alone has been shown to be approximately 60–70% [6,7] among patients with moderate IMC. Our group has also demonstrated that an early introduction of selective immunosuppressive therapy (SIT) is associated with favorable clinical outcomes in patients with IMC with a decreased need for steroid exposure, fewer steroid tapering attempts and fewer hospitalizations [8]. Robust data on the efficacy of the combination of SIT with budesonide in IMC therapy is lacking [29,30].

Another key finding in our analysis was that the duration of budesonide therapy in our sample was a median of 42.5 days, i.e., ~6 weeks, which is comparable, if not slightly better than the current practice with systemic steroids [9,31] which favors the use of the former given the gut-targeted effect and tolerable side-effect profile [29,30].

In our study population, the rate of developing diabetes, adrenal insufficiency or requiring long-term hydrocortisone after exposure to budesonide was low (2–9%) and less than what has been reported regarding systemic steroids as stated above. In regard to the two patients who developed diabetes after budesonide use: one was on chronic hydrocortisone and fludrocortisone for adrenal insufficiency and the other patient received a long course of prednisone and methylprednisolone for ICI pneumonitis. Moreover, with regards to diabetes and adrenal insufficiency, ICI use itself may serve as a confounding variable given its direct toxicity on the islet cells and adrenals [32,33,34]. The infection sustained after exposure to budesonide may not be reflective of a true adverse effect in an immunocompromised patient.

At the end of our study period, ~55% of the cohort had progression of their cancer and approximately 42% had stable disease. However, the majority of our sample had stage IV disease prior to exposure to ICI. Despite multiple confounding factors and the lack of high-quality prospective randomized data, some suggest a decreased efficacy of ICI and worse clinical outcomes characterized by decreased overall and progression-free survival in cancer patients on ICI with exposure to systemic immunosuppression with corticosteroids and biologics in these patients [35,36,37,38,39,40,41]. While this favors the use of a gut-targeting steroid with less bioavailability and systemic effects like budesonide, its outcomes on cancer specifically are yet to be studied.

Gut dysbiosis has been implicated in cases of IMC as well as cancer responsiveness to ICI and fecal microbiota transplant has been shown to be efficacious in the management of IMC refractory to the standard of care therapies [11,42,43]. The relationship between budesonide and the gut microbiome has not been well studied. There is some data to suggest that in patients with microscopic colitis, the gut microbiome differs from healthy individuals and budesonide treatment restores the gut microbiome to that similar of healthy individuals [44]. Larger studies to evaluate the effect of budesonide in conjunction with FMT on the gut microbiome may be beneficial to minimize immunosuppression in the cohort.

A small group of four patients in our cohort received budesonide as secondary prophylaxis for IMC and did well from a clinical standpoint. However, data in terms of using budesonide as a secondary prophylaxis to prevent recurrence is lacking; we do note that one clinical trial showed a lack of benefit of primary prophylaxis with budesonide in preventing IMC in patients with advanced melanoma treated with ipilimumab [45]. Further investigation on the use of budesonide as secondary prophylaxis for IMC especially upon ICI re-challenge can be a new area of interest in future clinical practice.

Our study is not without limitations. This is a retrospective study that was conducted at a tertiary cancer hospital and may not be representative of other types of clinical practice. Our overall sample size is small which limits the power of subgroup analyses. Additionally, approximately 1/3 of our patients were treated with budesonide after being on systemic steroids, so this could affect the actual impact of budesonide on those patients.

## 5. Conclusions

In conclusion, we observed remission rates for moderate IMC with budesonide alone that were comparable to systemic steroids. Long-term budesonide is associated with lower infection and complication risks than systemic steroids; it may also serve as a successful bridge from systemic corticosteroids and its use for secondary prophylaxis to avoid recurrent colitis remains yet to be explored. Larger prospective studies are necessary to determine the role of budesonide as well as its safety profile.

## Figures and Tables

**Table 1:** Patient characteristics, n = 69.

Characteristic	No. (%)
Age, years, median (IQR)	64 (52–71.5)
Sex, male	31 (44.9)
Race, white	53 (76.8)
Cancer type	
Melanoma	26 (37.6)
Genitourinary	11 (15.9)
Lung/head and neck	12 (17.3)
Gastrointestinal	6 (8.6)
Endocrine	3 (4.3)
Hematological	3 (4.3)
Gyn	4 (5.7)
Others	4 (5.7)
Cancer stage	
III	14 (20.2)
IV	55 (79.7)
Type of ICI	
Anti–CTLA-4 monotherapy	7 (10.1)
Anti–PD-1/L1 monotherapy	28 (40.5)
Combination anti–CTLA-4 and anti–PD-1/L1	34 (49.3)
Cycles of ICI, median (IQR)	3 (2–6)
Active treatment with other chemotherapy	
Continued with ICI	22 (31.8)
Continued with other chemotherapy	12 (17.3)
Median time duration of ICI therapy, months (IQR)	2 (0–0.5)
Median time duration from gastrointestinal irAE diagnosis and first dose of ICI, days (IQR)	95 (33–323)

Abbreviations: CTLA-4, cytotoxic T lymphocyte antigen 4; ICI, immune checkpoint inhibitor; IQR, interquartile range; PD-1/PD-L1, programmed cell death 1/programmed death ligand 1; irAE, immune-related adverse event.

**Table 2:** Characteristics of gastrointestinal irAE in patients with colorectal cancer.

	Diagnosed with Colitis, n = 69	Budesonide-Treated Group, n = 38
Symptoms	No. (%)	No. (%)
Diarrhea	69 (100)	38 (100)
Abdominal pain	65 (94.2)	38 (100)
Median fecal calprotectin before treatment, μg/mg (IQR)	671 (106.9–1000)	583 (171–981)
Median CTCAE grade of colitis (IQR)	2 (1–2)	2 (1–2)
Median CTCAE grade of diarrhea (IQR)	3 (2–3)	3 (2–3)
Hospitalization required	35 (50.7)	23 (60.5)
All-cause mortality	26 (37.6)	17 (44.7)

Abbreviations: CTCAE v5, Common Terminology Criteria for Adverse Events version 5; ICI, immune checkpoint inhibitor; IMC, immune-mediated colitis; IQR, interquartile range; TNF, tumor necrosis factor; FMT, fecal microbiota transplantation; irAE, immune-related adverse event.

**Table 3:** Endoscopy-related characteristics for patients diagnosed with colitis.

At the Time of Colitis Diagnosis	Diagnosed with Colitis, n = 50,	Budesonide-Treated Group, n = 28
Endoscopic findings (only those that underwent endoscopy), n = 50	No. (%)	No. (%)
Ulcers	9 (18)	5 (17.8)
Non-ulcer inflammation	32 (64)	19 (67.8)
Normal	9 (18)	4 (14.2)
Histologic findings		
Active inflammation	39 (78)	22 (78.5)
Chronic inflammation	4 (8)	2 (7.1)
Microscopic colitis	2 (4)	0 (0)
Normal	5 (10)	3 (10.7)
Treatment of IMC, n = 69		
Budesonide alone	38 (55)	38 (100)
Budesonide plus infliximab only	7 (10.1)	-
Budesonide plus vedolizumab only	15 (21.7)	-
Budesonide plus both infliximab and vedolizumab	5 (7.2)	-
Budesonide plus ustekinumab add-on	4 (5.7)	-
FMT	3 (4.3)	-
Complications of IMC	2 (2.8)	

Abbreviations: IMC, immune-mediated colitis; FMT, fecal microbiota transplantation.

**Table 4:** Budesonide use characteristics and outcome, n = 69.

Type of Use in Regards of Budesonide	No. (%)
Primarily to treat IMC	39 (56.5)
Bridged from systemic steroid	23 (33.3)
Prophylactic use	4 (5.7)
Other use	3 (4.3)
Budesonide use	
Median time of budesonide use, days	42.5 (28–107)
Outcomes of colitis	
Remission	52 (75.3)
Recurrence	17 (24.6)

Abbreviations: IMC, immune-mediated colitis.

**Table 5:** Budesonide safety profile, n = 69.

Adverse Event	No. (%)
Patients with Diabetes prior to budesonide use	12 (17.3)
New Onset Diabetes after budesonide (n = 57)	2 (3.5)
Infection within 2 months after budesonide	5 (7.2)
GU/UTI infection	2 (2.9)
Pneumonia infection	3 (4.3)
Adrenal Insufficiency or need for maintenance low-dose hydrocortisone after budesonide	6 (8.7)
ICU stay after budesonide	2 (2.9)
Cancer Status after budesonide at the time of last follow up	
Stable	29 (42)
Progression	38 (55)
Remission	2 (2.9)

Abbreviations: GU, genitourinary; UTI, urine tract infection; ICU, intensive care unit.

**Table 6:** Safety profile of budesonide-treated groups, n = 62.

	Primarily to Treat IMC, n = 23	Bridged from Systemic Steroid, n = 39
Adverse Event	No. (%)	No. (%)
New Onset Diabetes after budesonide	2 (8.6)	0 (0)
Infection within 2 months after budesonide	2 (8.6)	3 (7.6)
GU/UTI infection	1 (4.3)	1 (2.56)
Pneumonia infection	1 (4.3)	2 (5.1)
Adrenal Insufficiency or need for maintenance low-dose hydrocortisone after budesonide	2 (8.6)	4 (10.2)
ICU stay after budesonide	0 (0)	2 (5.1)

Abbreviations: GU, genitourinary; UTI, urine tract infection; ICU, intensive care unit.

## Data Availability

The data presented in this study are available on request from the corresponding author due to privacy/ethical restrictions.

## Supplementary Materials

The following supporting information can be downloaded at: https://www.mdpi.com/article/10.3390/cancers16101919/s1. Figure S1: Endoscopy images demonstrating: (a) high-risk features, (b) low-risk features, (c) Ulcerative colitis like disease, (d) Crohn’s like disease; yellow arrow demonstrates large deep mucosal ulceration surrounded by normal mucosa.; Figure S2: Histopathology images demonstrating: (a) colonic mucosa with architecture distortion, basal plasmacytosis (white arrow), cryptitis (yellow arrow) and crypt abscess (red arrow), (b) Colonic mucosa with mild architecture distortion and minimal features of active inflammation.
