# Supplementary material for: Outcomes of Budesonide as a Treatment Option for Immune Checkpoint Inhibitor-Related Colitis in Patients with Cancer
Source: Cancers (Basel). 2024 May 18;16(10):1919. doi: 10.3390/cancers16101919 (PMC11120342; doi:10.3390/cancers16101919)

**Figure S1.** Endoscopy images demonstrating: (a) high-risk features, (b) low-risk features, (c) Ulcerative colitis like disease, (d) Crohn's like disease; yellow arrow demonstrates large deep mucosal ulceration surrounded by normal mucosa.

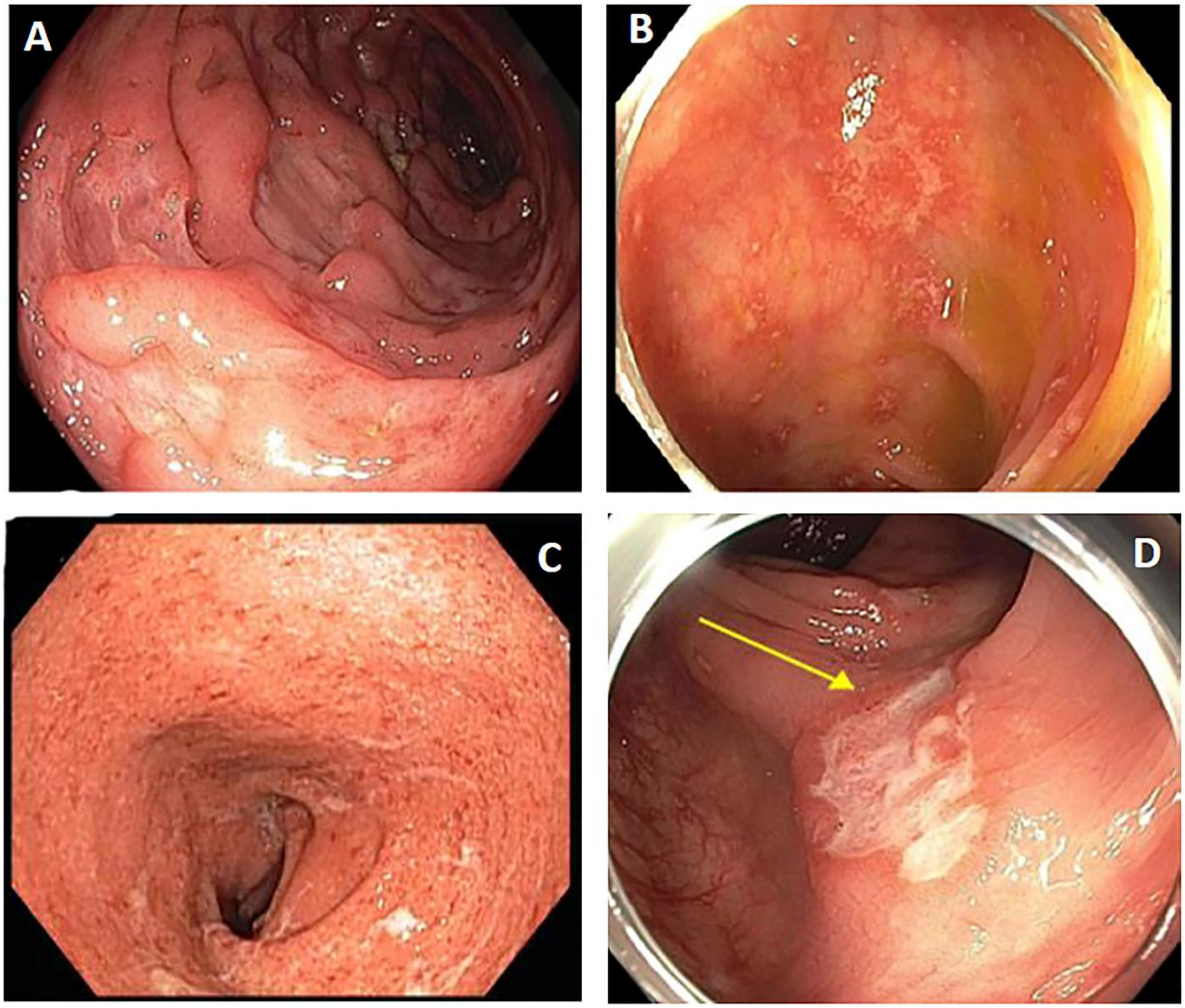

**Figure S2.** Histopathology images demonstrating: (a) colonic mucosa with architecture distortion, basal plasmacytosis (white arrow), cryptitis (yellow arrow) and crypt abscess (red arrow), (b) Colonic mucosa with mild architecture distortion and minimal features of active inflammation.

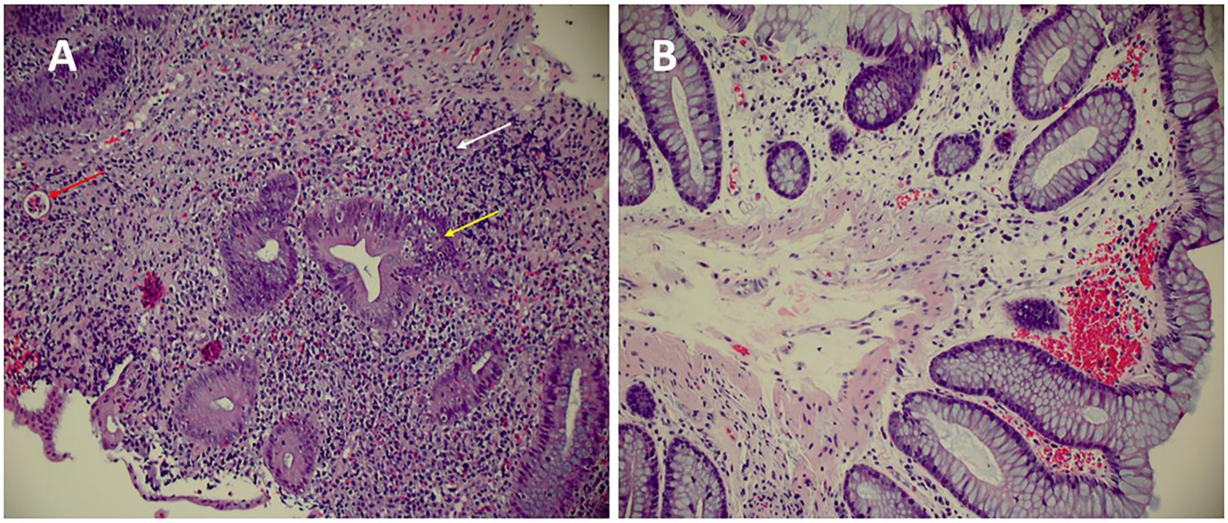

Supplement: Supplementary file 1 [file cancers-16-01919-s001.zip › cancers-2969618-supplementary.pdf]
